# Physiological response to prone positioning in intubated adults with COVID-19 acute respiratory distress syndrome: a retrospective study

**DOI:** 10.1186/s12931-022-02247-8

**Published:** 2022-11-19

**Authors:** Andrea Boffi, Maximilien Ravenel, Ermes Lupieri, Antoine Schneider, Lucas Liaudet, Michel Gonzalez, Jean-Daniel Chiche, Lise Piquilloud

**Affiliations:** 1 Adult Intensive Care Unit, Lausanne University Hospital, Lausanne, Switzerland; 2 Faculty of Biology and Medicine, University of Lausanne, Lausanne, Switzerland; 3 Department of Thoracic Surgery, Lausanne University Hospital, Lausanne, Switzerland

**Keywords:** ARDS, COVID-19, Proning, Prone position, Alveolo-arterial gradient, Oxygenation, Ventilatory ratio, Dead space, Respiratory system compliance

## Abstract

**Background:**

COVID-19 related acute respiratory distress syndrome (ARDS) has specific characteristics compared to ARDS in other populations. Proning is recommended by analogy with other forms of ARDS, but few data are available regarding its physiological effects in this population. This study aimed to assess the effects of proning on oxygenation parameters (PaO_2_/FiO_2_ and alveolo-arterial gradient (Aa-gradient)), blood gas analysis, ventilatory ratio (VR), respiratory system compliance (C_RS_) and estimated dead space fraction (V_D_/V_T_ HB). We also looked for variables associated with treatment failure.

**Methods:**

Retrospective monocentric study of intubated COVID-19 ARDS patients managed with an early intubation, low to moderate positive end-expiratory pressure and early proning strategy hospitalized from March 6 to April 30 2020. Blood gas analysis, PaO_2_/FiO_2_, Aa-gradient, VR, C_RS_ and V_D_/V_T_ HB were compared before and at the end of each proning session with paired t-tests or Wilcoxon tests (p < 0.05 considered as significant). Proportions were assessed using Fischer exact test or Chi square test.

**Results:**

Forty-two patients were included for a total of 191 proning sessions, median duration of 16 (5–36) hours. Considering all sessions, PaO_2_/FiO_2_ increased (180 [148–210] vs 107 [90–129] mmHg, p < 0.001) and Aa-gradient decreased (127 [92–176] vs 275 [211–334] mmHg, p < 0.001) with proning. C_RS_ (36.2 [30.0–41.8] vs 32.2 [27.5–40.9] ml/cmH_2_O, p = 0.003), VR (2.4 [2.0–2.9] vs 2.3 [1.9–2.8], p = 0.028) and V_D_/V_T_ HB (0.72 [0.67–0.76] vs 0.71 [0.65–0.76], p = 0.022) slightly increased. Considering the first proning session, PaO_2_/FiO_2_ increased (186 [165–215] vs 104 [94–126] mmHg, p < 0.001) and Aa-gradient decreased (121 [89–160] vs 276 [238–321] mmHg, p < 0.001), while C_RS_, VR and V_D_/V_T_ HB were unchanged. Similar variations were observed during the subsequent proning sessions. Among the patients who experienced treatment failure (defined as ICU death or need for extracorporeal membrane oxygenation), fewer expressed a positive response in terms of oxygenation (defined as increase of more than 20% in PaO_2_/FiO_2_) to the first proning (67 vs 97%, p = 0.020).

**Conclusion:**

Proning in COVID-19 ARDS intubated patients led to an increase in PaO_2_/FiO_2_ and a decrease in Aa-gradient if we consider all the sessions together, the first one or the 4 subsequent sessions independently. When considering all sessions, C_RS_ increased and VR and V_D_/V_T_ HB only slightly increased.

**Supplementary Information:**

The online version contains supplementary material available at 10.1186/s12931-022-02247-8.

## Background

Coronavirus Disease 19 (COVID-19) pandemic is still causing thousands of deaths worldwide [1]. Between 5 and 34% of hospitalized patients develop severe disease and are admitted in the intensive care unit (ICU). Most of these patients fulfill the criteria for acute respiratory distress syndrome (ARDS), with a high mortality rate [2–8]. Prone positioning demonstrated physiological and survival benefits in moderate to severe ARDS non related to COVID-19 and is recommended by the current international guidelines as part of ARDS management [9–12]. The prone position improves oxygenation by promoting dorsal recruitment, allowing for a more homogeneous ventilation distribution, and improving ventilation/perfusion matching. It may also reduce lung stress and strain with a potential for reducing Ventilation Induced Lung Injury (VILI) [13]. It is still debated if COVID-19 related ARDS represents a distinct entity and whether common treatments used in ARDS are equally effective [14–16]. International guidelines [17, 18] and experts [19], however, recommend the use of prone position in patients with COVID-19 related moderate to severe ARDS and this technique is more widely used than in the past [20, 21]. Some reports suggest a benefit of proning in COVID-19 patients [21–29], but few data are available on the physiological effects of proning in COVID-19 ARDS, except for the effect on the PaO_2_/FiO_2_ ratio. Factors associated with success and failure have also not been extensively described.

The main aim of this study was to assess the effects of proning in COVID-19 ARDS patients on oxygenation parameters (PaO_2_/FiO_2_ and alveolar-arterial gradient—Aa-gradient—) both for all the proning sessions considered together and for the first five proning sessions. As a secondary aim, we assessed the effects of proning (all sessions and first to fifth sessions) on other physiological and respiratory parameters such as respiratory mechanics, and dead space fraction estimates. Finally, as an ancillary aim, we looked for factors associated with treatment failure defined as ICU death or the need for veno-venous extracorporeal membrane oxygenation (VV-ECMO).

## Methods

Retrospective study performed in the adult intensive care unit of the Lausanne University Hospital, Switzerland. The study protocol was accepted by the “Commission cantonale d’éthique de la recherche sur l’être humain” CER-VD (protocol number 2020-01453). All consecutive patients admitted during the first pandemic wave between the 06th of March and the 30th of April 2020 for PCR confirmed COVID-19 ARDS [30] who were invasively ventilated and proned at least once during their ICU stay were considered for inclusion. Patients proned only during ECMO treatment, patients already proned in a referring hospital and patients who refused utilization of their clinical data were excluded. To note, in Switzerland, for a retrospective analysis, the patients must accept the use of their recorded data for research purposes. Waiver of consent is possible, if accepted by the Ethics committee, for the deceased patients who did not refuse the use of their data before death, for the patients who did not adopt a position on the general consent procedure 6 weeks after having been contacted twice and for the patients who could not be contacted.

During the study period, all COVID-19 ARDS patients were treated following the local written clinical protocol that described intubation criteria, ventilation strategies including positive end-expiratory pressure (PEEP) setting (low to moderate PEEP strategy, clinical protocol available in the electronic supplementary material) and indications for proning. According to this protocol, all intubated patients with a PaO_2_/FiO_2_ < 150 mmHg and a FiO_2_ > 0.6 after curarisation were proned. When proning was decided, at least three sessions were performed, unless contraindicated or poorly tolerated. Data used in this study were retrieved from the patients’ medical file and the electronic medical records system. We collected data on demographics, established risk factors for severe COVID-19 infection, severity scores (SAPS II, SOFA) and blood gas analysis at ICU admission. On the day of intubation, we collected SOFA score, first blood gas analysis after intubation and concomitant end-tidal CO_2_ (EtCO_2_), ventilatory settings, plateau pressure and compliance of the respiratory system  (C_RS_). Time from admission to intubation, from intubation to first proning and from the first documented PaO_2_/FiO_2_ < 150 mmHg to the first proning were recorded.

For each proning session we collected its duration and related serious complications (tube or catheter displacement, tube obstruction, cardiorespiratory arrest). Blood gas analysis and concomitant EtCO_2_, ventilatory settings, plateau pressure (Pplat), driving pressure (∆P) defined as Pplat–PEEP, and C_RS_ were recorded within two hours before placement in prone position (pre-PP) and within two hours before returning supine (end-PP). Heart rate (HR), mean arterial pressure (MAP) and dose of norepinephrin in µg/kg/min were also recorded at the time of the blood gas analyses (pre-PP and end-PP times). As outcome data we recorded the number of days under mechanical ventilation, the number of ventilator free days at day 28 after intubation and the ICU and hospital length of stay.

Based on the collected data, we calculated for pre-PP and for end-PP time points Aa-gradient, PaCO_2_–EtCO_2_ gradient, ventilatory ratio (VR) [31] and dead space fraction estimated using the unadjusted Harris-Benedict estimate of resting energy expenditure and the rearranged Weir equation for CO_2_ production (V_D_/V_T_ HB) [32] (Equations used are provided in Table 1).

**Table 1:** Summary of formulas

$Aa-gradient$	$\left[Fi{O}_{2} \times \left([Patm-P{H}_{2}O\right)\right]-\left(\frac{PaC{O}_{2}}{RQ}\right)-Pa{O}_{2}$
$VR$	$\frac{\left[{\dot{V}}_{E} \times PaC{O}_{2}\right]}{PBW x 100 x 37.5}$
$PBW$	$X+\left[0.91 \times \left(H-152.4\right)\right]$ $X=50 in men, X=45.5 in women$
$\frac{{V}_{D}}{{V}_{T}} HB$	$1- \frac{(0.863 x \dot{V}C{O}_{2}}{({\dot{V}}_{E} \times PaC{O}_{2})}$
$\dot{V}C{O}_{2}$	$\frac{REE}{\left(\frac{5.616}{RQ}+1.584\right)}$
$REE for males$	$66.473+13.752 \times W+5.003 \times H-6.755 \times Y$
$REE for females$	$655.096+9.563 \times W+1.850 \times H-4.676\times Y$

*Aa-gradient*: alveolo-arterial gradient of O_2_, in mmHg; *FiO*_*2*_: fraction of inspired oxygen; *Patm*: atmospheric pressure, in mmHg, 715 mm Hg in Lausanne; *PH*_*2*_*O*: vapor pressure, in mmHg, approximated to 47 mmHg; *PaCO*_*2*_: arterial partial pressure of carbon dioxide, in mmHg; *RQ*: respiratory quotient, approximated to 0.8; *PaO*_*2*_: arterial partial pressure of oxygen, in mmHg; *VR*: ventilatory ratio, adimensional; *⩒*_*E*_: minute ventilation in ml/min; *PBW*, predicted body weight in kg; *V*_*D*_*/V*_*T*_* HB:* dead space fraction estimated using the unadjusted Harris-Benedict estimate of resting energy expenditure and the rearranged Weir equation for CO_2_ production; *REE*: resting energy expenditure; *⩒CO*_*2*_: carbon dioxide production, in ml/min; *H*: height in cm; *W*: weight in kg

As primary study outcome, we analyzed the effects of proning on oxygenation parameters (PaO_2_/FiO_2_ and Aa-gradient) both for all the prone positioning sessions and for the first to the fifth sessions independently. As a secondary aim we analyzed the effects of proning on other physiological and respiratory variables (blood gas analysis, VR, V_D_/V_T_ HB, Pplat, ΔP and C_RS_) also for all the sessions and for the first to the fifth sessions. Positive response in terms of oxygenation (patients considered as O_2_-responders) was arbitrarily defined, as in a previous study [24], as an increase of more than 20% in PaO_2_/FiO_2_. Significant CO_2_ clearance (patients considered as CO_2_-responders) was defined as a decrease in PaCO_2_ of 1 mmHg or more according to the study by Gattinoni et al. [33] that showed a correlation between decrease in PaCO_2_ and outcome in non-COVID ARDS patients who were proned. To identify variables associated with patients’ outcomes, we arbitrarily defined treatment failure as a composite outcome including ICU death and need of VV-ECMO support. Consequently, treatment success was considered as ICU survival without VV- ECMO.

### Statistics

Comparison between values before and at the end of proning (pre-PP and for end-PP timepoints) were assessed by paired T test or Wilcoxon signed rank test, according to data distribution. Continuous data for responders and non-responders and for treatment success and failure were compared with unpaired t test or Mann Whitney U test according to their distribution. Proportions were assessed using Fischer exact test or Chi square test as appropriate. Because most data were not normally distributed (Shapiro Wilk test), data are presented as medians with first and third quartiles (Q1-Q3). Data were analyzed with GraphPad Prism version 9.1 (GraphPad Software, San Diego, CA).

## Results

**Fig. 1:**
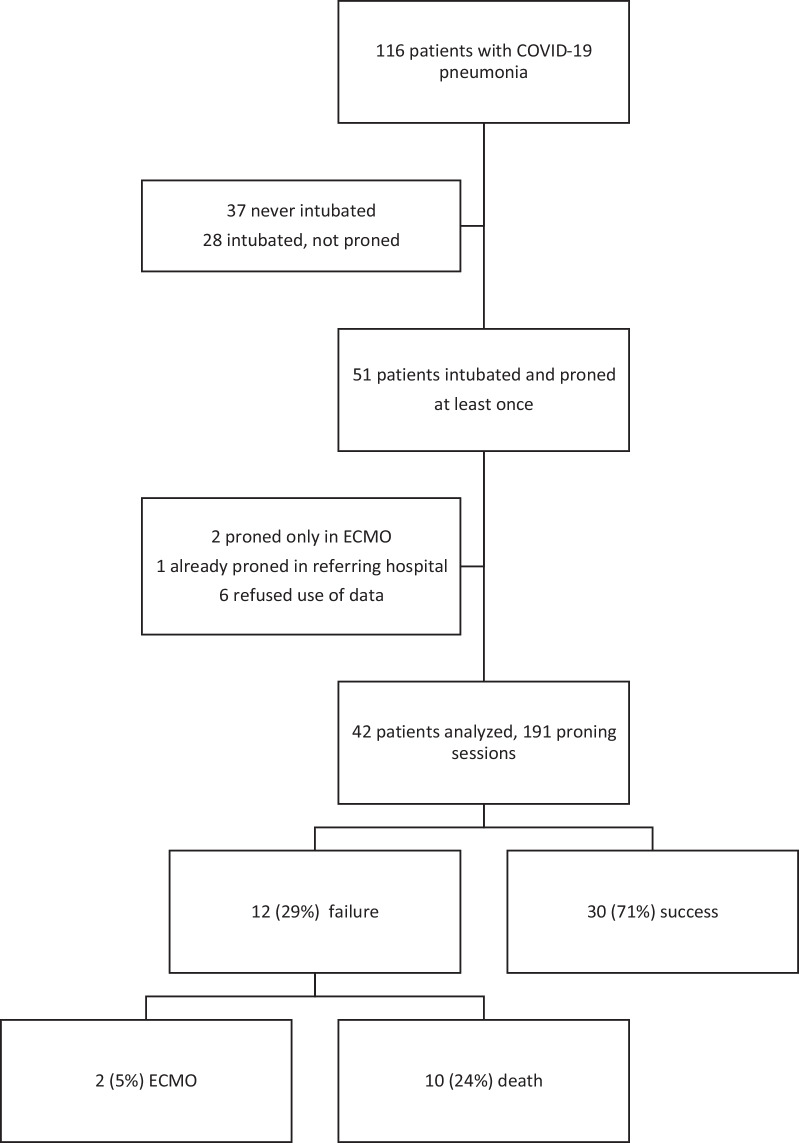
Flowchart

A total of 116 patients were admitted in our ICU due to COVID-19 pneumonia during the study period. Fifty-one were invasively ventilated and proned. Nine patients were excluded from the final analysis, 2 because they were proned during VV-ECMO treatment only, 6 because they did not accept the use of their data for retrospective analysis and 1 because he/she was already proned in another hospital before admission in our ICU. Thus, 42 patients were included in the final analysis (Fig. 1, flowchart)*.* A total of 191 sessions were performed in these 42 patients and analyzed to assess the effect of proning on physiological parameters.

### Baseline population and characteristics

The baseline characteristics and the baseline physiological and ventilatory parameters within 2 h after intubation (first available blood gas analysis after intubation, all patients paralyzed) are mentioned in Table 2 for the general patients population and for patients who experienced treatment success and failure. According to the Berlin classification [30], shortly after intubation among the global patients population, 10% were classified as severe ARDS, 80% as moderate and 10% as mild.

**Table 2:** Baseline general characteristics, baseline physiological and ventilatory parameters within 2 h after intubation and outcome data

	Overall (n = 42)	Success (n = 30)	Failure (n = 12)	P value
*Baseline characteristics*				
Age	63 (57–72)	60 (56–69)	73 (61–78)	0.009
Male (%)	31 (74)	22 (73)	9 (75)	0.99
Weight, kg	85 (72–105)	93 (74–115)	79 (71–86)	0.03
Height, m	1.75 (1.67–1.80)	1.75 (1.67–1.80)	1.75 (1.66–1.76)	0.41
BMI, kg/m^2^	27.7 (25.4–33.7)	28.6 (26.0–37.1)	27.3 (25.1–28.2)	0.19
SAPS II	41 (34–46)	38 (33–46)	45 (38–48)	0.11
SOFA admission	3 (2–5)	3 (2–4)	6 (3–8)	0.01
SOFA intubation	7 (6–8)	7 (6–8)	7 (6–9)	0.61
Severity risk factor (%)	31 (74)	22 (73)	9 (75)	0.99
Hypertension, n (%)	22 (52)	15 (50)	7 (58)	0.74
Diabetes mellitus, n (%)	7 (17)	6 (20)	1 (8)	0.65
Cardiovascular disease, n (%)	7 (17)	4 (13)	3 (25)	0.39
Cancer, n (%)	3 (7)	3 (10)	0 (0)	0.55
Chronic respiratory disease n (%)	11 (26)	8 (27)	3 (25)	0.99
Chronic kidney disease, n (%)	3 (7)	3 (10)	0 (0)	0.55
BMI > 30, n (%)	15 (36)	14 (47)	1 (8)	0.03
Smokers, n (%)	10 (24)	10 (33)	0 (0)	0.04
Immunodeficiency, n (%)	0 (0)	0 (0)	0 (0)	0.99
*Physiological variables*				
PaO_2_, mmHg	83 (76–100)	82 (75–99)	89 (83–111)	0.20
FiO_2_	0.60 (0.50–0.80)	0.60 (0.56–0.80)	0.50 (0.45–0.60)	0.21
PaO_2_/FiO_2_, mmHg	137 (118–172)	133 (118–155)	178 (145–197)	0.05
SaO_2_, %	95 (93–96)	94 (93–96)	95 (93–97)	0.82
pH	7.30 (7.22–7.34)	7.28 (7.19–7.32)	7.31 (7.24–7.36)	0.39
PaCO_2_, mmHg	50 (43–58)	52 (45 -58)	46 (42–58)	0.51
HCO_3_^−^, mmol/L	23.3 (21.4–24.4)	23.6 (21.9–25.9)	22.2 (20.0–23.1)	0.01
Hb, g/L	127 (110–135)	125 (111–135)	128 (109–136)	0.65
Lactate, mmol/L	1.1 (0.9–1.3)	1.1 (0.8–1.3)	1.2 (0.9–1.5)	0.63
EtCO_2_ mmHg	42 (37–48)	42 (37–47)	43 (34–52)	0.83
PaCO_2_-EtCO_2_ mmHg	7 (1–11)	5 (1–16)	7 (2–10)	0.93
Aa-gradient, mmHg	255 (200–345)	265 (241–368)	172 (164–254)	0.04
VR	1.7 (1.3–2.2)	1.7 (1.2–2.2)	1.8 (1.4–2.4)	0.59
V_D_/V_T_ HB	0.59 (0.51–0.68)	0.58 (0.50–0.68)	0.65 0.56–0.73)	0.15
*Ventilatory data*				
Vt/PBW, ml/kg	6.7 (6.0–7.0)	6.6 (6.1–7.0)	6.8 (6.0–6.9)	0.85
RR, min^−1^	20 (17–22)	18 (16–22)	22 (20–24)	0.13
PEEP, cmH_2_O	12 (10–14)	13 (10–14)	12 (10–12)	0.09
Pplat, cmH_2_O	25 (22–28)	25 (22–28)	22 (21–27)	0.23
∆P, cmH_2_O	13 (10–14)	13 (10–14)	12 (10–14)	0.65
C_RS_, ml/cmH_2_O	36.4 (29.3–42.5)	36.4 (30.0–40.0)	36.1 (28.5–44.0)	0.93
*Hemodynamics data*				
HR, min^−1^	87 (70–100)	84 (73–108)	89 (65–99)	0.41
MAP, mmHg	74 (67–84)	73 (68–82)	76 (66–86)	0.48
Norepinephrine, µcg/kg/min	0 (0–0.07)	0 (0–0.06)	0.02 (0–0.1)	0.41
*Outcome data*				
LoS Hospital, days	29 (23–40)	29 (24–40)	24 (12–29)	0.06
LoS ICU, days	18 (13–27)	18 (13–25)	23 (10–29)	0.97
Ventilation days	15 (10–20)	14 (11–17)	20 (9–24)	0.24
Ventilator free days at Day 28	11 (0–17)	14 (11–17)	0 (0–1)	< 0.001

*BMI*: Body Mass Index, *SAPS II*: Simplified Acute Physiology Score II, *SOFA*: Sequential Organ Failure Assessment, *PaO*_*2*_: arterial partial pressure of oxygen, *FiO*_*2*_: fraction of inspired oxygen, *SaO*_*2*_: arterial oxygen saturation, *PaCO*_*2*_: partial arterial pressure of carbon dioxide, *HCO*_*3*_^−^: bicarbonate, *Hb*: hemoglobin, *EtCO*_*2*_: end tidal CO_2,_ *Aa-gradient*: alveolo arterial gradient, *VR*: ventilatory ratio, *V*_*D*_*/V*_*T*_* HB*: dead space fraction estimated using the unadjusted Harris-Benedict estimate of resting energy expenditure and the rearranged Weir equation for CO_2_ production, *Vt*: tidal volume, *PBW*: predicted body weight, *RR*: respiratory rate, *Pplat*: plateau pressure, *PEEP*: positive end expiratory pressure, *∆P*: driving pressure, *C*_*RS*_: compliance of the respiratory system, *HR*: heart rate, *MAP*: mean arterial pressure, *LoS*: length of stay, p values refer to the comparison between the treatment success and failure groups

Among the 42 included patients, 12 (29%) experienced treatment failure, of whom 10 (24%) died in the ICU. Two (5%) eventually needed VV-ECMO support. These 2 patients were successfully weaned from VV-ECMO and mechanical ventilation and discharged from the hospital. The outcomes of the general population and of the patients who experienced treatment success and failure are mentioned in Table 2.

### General data about intubation and proning

Median time from ICU admission to intubation was 4 (1–16) hours for the 36 patients who were not already intubated at ICU admission. Time from intubation to first proning was 46 (13–90) hours and time from the first PaO_2_/FiO_2_ < 150 mmHg to proning was 16 (5–36) hours. Patients sustained a median of 3 (2–6) proning sessions, with a median duration of 17 (16–19) hours. No serious complications attributed to prone positioning occurred during the 191 analyzed sessions.

No statistically significant differences between the success and failure groups were noted in intubation timing (p = 0.11) and proning timings (intubation to proning, p = 0.23 and first PaO_2_/FiO_2_ < 150 mmHg to proning, p = 0.48). The number of proning sessions (p = 0.94) or their duration (p = 0.39) were also not different between the 2 groups.

### Effect of proning on physiological and respiratory data

**Fig. 2:**
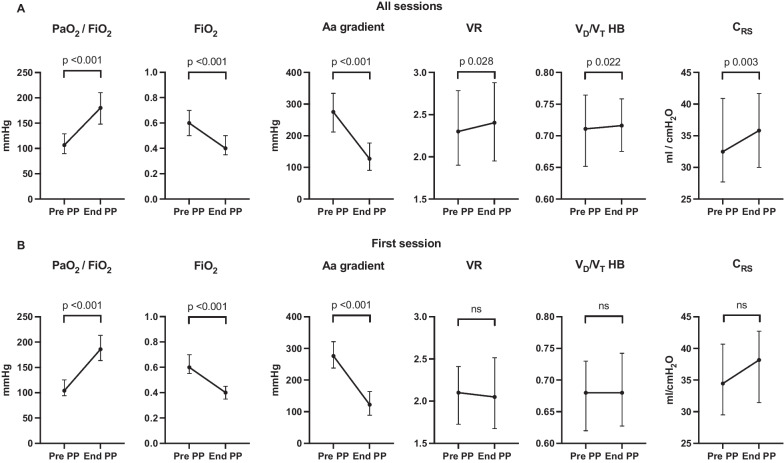
Variations in PaO_2_/FiO_2_, FiO_2_, Aa gradient, C_RS_, VR and V_D_/V_T_ HB for all sessions (**A**) and first session (**B**) before (pre PP) and at the end (end-PP) of the prone position session. *PaO*_*2*_: arterial partial pressure of oxygen, *FiO*_2_: fraction of inspired oxygen, *Aa gradient*: alveolo-arterial gradient, *VR*: ventilatory ratio, *V*_*D*_*/V*_*T*_* HB*: dead space fraction estimated using the unadjusted Harris-Benedict estimate of resting energy expenditure and the rearranged Weir equation for CO_2_ production, *C*_*RS*_: Compliance of the respiratory system

Figure 2A illustrates the changes in PaO_2_/FiO_2_, FiO_2_, Aa-gradient, VR and V_D_/V_T_ HB and C_RS_ for all proning sessions considered together, between pre-PP and end-PP time points. The time between pre-PP blood gas analysis and start of proning was 66 (42–113) minutes. The time between end-PP blood gas analysis and end of proning was 72 (32–115) minutes. Figure 2B illustrates the changes on the same parameters for the first proning session.

Additional physiological and respiratory variables, including hemodynamic data, before and at the end of the proning sessions are presented in Additional file 1 (Table S1) for all proning sessions together and for the first to the fifth sessions. Looking at the response in terms of oxygenation, 83% of all proning sessions led to an increase of more than 20% in the PaO_2_/FiO_2_ ratio (O_2_ responders). Only 44% of all proning sessions led to a significant reduction in CO_2_.

Table 3 illustrates the effects of the first to the third proning sessions on the changes in PaO_2_/FiO_2_, FiO_2_, Aa-gradient, VR, V_D_/V_T_ HB, Pplat, ΔP and C_RS_ for all the patients and for the patients who experienced treatment success or failure. The percentage of O_2_ and CO_2_ responders for the first to the third proning sessions for each patient group are also mentioned in Table 3. p values in Table 3 refer to the comparison between the treatment success and treatment failure groups. Additional file 1 (Table S2) mentions the same information for the fourth and the fifth sessions.

**Table 3:** Variations in physiological and ventilatory parameters during the first (n = 42), the second (n = 37) and the third (n = 31) proning sessions

	Overall	Success	Failure	p value
*Variation in PaO*_*2*_*/FiO*_*2*_*, mmHg, from pre-PP to end-PP*				
1st pronation	+ 75 (+ 54 to + 114)	+ 86 (+ 66 to + 125)	+ 58 (+ 10 to + 73)	0.001
2nd pronation	+ 69 (+ 33 to + 123)	+ 74 (+ 34 to + 126)	+ 50 (+ 8 to + 117)	0.53
3rd pronation	+ 48 (+ 24 to + 68)	+ 52 (+ 16 to + 74)	+ 45 (+ 26 to + 69)	0.80
*Variation in FiO*_*2*_*, from pre-PP to end-PP*				
1st pronation	− 0.20 (− 0.33 to − 0.15)	− 0.25 (− 0.38 to − 0.17)	− 0.18 (− 0.24 to − 0.02)	0.02
2nd pronation	− 0.10 (− 0.30 to − 0.05)	− 0.10 (− 0.30 to − 0.05)	− 0.10 (− 0.31 to + 0.04)	0.68
3rd pronation	− 0.10 (− 0.20 to − 0.05)	− 0.10 (− 0.20 to − 0.03)	− 0.15 (− 0.27 to − 0.05)	0.50
*Variation in Aa-gradient, mmHg, from pre-PP to end-PP*				
1st pronation	− 160 (− 232 to − 100)	− 170 (− 248 to − 124)	− 105 (− 169 to − 13)	0.01
2nd pronation	− 98 (− 205 to − 34)	− 98 (− 214 to − 40)	− 98 (− 194 to + 21)	0.68
3rd pronation	− 85 (− 138 to − 41)	− 85 (− 132 to − 9)	− 95 (− 187 to − 52)	0.45
*O*_*2*_* responders*				
1st pronation	37/42 (88%)	29/30 (97%)	8/12 (67%)	0.002
2nd pronation	29/37 (78%)	22/27 (82%)	7/10 (70%)	0.66
3rd pronation	22/31 (71%)	17/25 (68%)	5/6 (83%)	0.64
*Variation in VR, from pre-PP to end-PP*				
1st pronation	0.02 (− 0.28 to 0.21)	− 0.13 (− 0.34 to + 0.13)	+ 0.13 (− 0.14 to + 0.22)	0.21
2nd pronation	+ 0.08 (− 0.17 to + 0.28)	+ 0.07 (− 0.16 to + 0.29)	+ 0.09 (− 0.21 to + 0.40(	0.72
3rd pronation	+ 0.27 (− 0.08 to + 0.37)	+ 0.25 (− 0.06 to + 0.40)	+ 0.27 (− 0.14 to + 0.33)	0.85
*Variation in V*_*D*_*/V*_*T*_* HB, from pre-PP to end-PP*				
1st pronation	+ 0.01 (− 0.03 to 0.02)	− 0.01 (− 0.08 to + 0.02)	0.01 (− 0.02 to + 0.03)	0.19
2nd pronation	+ 0.01 (− 0.04 to + 0.04)	+ 0.02 (− 0.03 to + 0.03)	+ 0.01 (− 0.04 to + 0.06)	0.86
3rd pronation	+ 0.04 (− 0.01 to + 0.06)	+ 0.04 (− 0.01 to + 0.07)	+ 0.02 (− 0.01 to + 0.06)	0.77
*CO*_*2*_* responders*				
1st pronation	21/42 (50%)	16/30 (53%)	5/12 (42%)	0.51
2nd pronation	14/37 (38%)	9/27 (33%)	5/10 (50%)	0.45
3rd pronation	9/31 (29%)	7/25 (28%)	2/6 (33%)	0.77
*Variation in PEEP, cmH*_*2*_*O, from pre-PP to end-PP*				
1st pronation	0 (0 to 0)	0 (− 1 to 0)	0 (0–0)	0.99
2nd pronation	0 (− 2 to 0)	0 (− 2 to 0)	0 (− 2 to 0)	0.95
3rd pronation	0 (− 1 to 0)	0 (− 1 to 0)	+ 1 (0 to + 2)	0.08
*Variation in Pplat, cmH*_*2*_*O, from pre-PP to end-PP*				
1st pronation	− 1 (− 3 to + 1)	− 1 (− 3 to + 1)	1 (− 1 to + 2)	0.11
2nd pronation	− 1 (− 3 to 0)	− 2 (− 4 to 0)	+ 2 (0 to + 6)	< 0.001
3rd pronation	− 1 (− 2 to + 1)	− 1 (− 2 to 0)	0 (− 1 to + 4)	0.09
*Variation in ∆P, cmH*_*2*_*O, from pre-PP to end-PP*				
1st pronation	− 1 (− 2 to + 1)	− 1 (− 3 to + 1)	1 (− 1 to + 3)	0.14
2nd pronation	− 1 (− 2 to 0)	− 1 (− 3 to 0)	+ 3 (+ 2 to + 6)	< 0.001
3rd pronation	0 (− 2 to + 1)	0 (− 2 to + 1)	0 (− 1 to + 2)	0.42
*Variation in C*_*RS*_*, ml/ cmH*_*2*_*O, from pre-PP to end-PP*				
1st pronation	+ 2.7 (− 2.4 to + 7.3)	+ 3.8 (− 2.1 to + 8.2)	− 0.5 (− 6.3 to + 3.8)	0.13
2nd pronation	+ 2.3 (− 0.9 to + 7.8)	+ 3.9 (0 to + 8.2)	− 6.3 (− 14.1 to − 1.6)	< 0.001
3rd pronation	+ 0.8 (− 3.2 to + 5.4)	+ 1.7 (− 3.4 to + 5.4)	0 (− 3.3 to 5.7)	0.89

Comparisons between the success and failure group, *PaO*_*2*_: arterial partial pressure of oxygen, *FiO*_*2*_: fraction of inspired oxygen, *Aa-gradient*: alveolo-arterial gradient, *O*_*2*_* responders*: patients presenting a 20% increase in PaO_2_/ FiO_2_ during proning, *VR*: ventilatory ratio, *V*_*D*_*/V*_*T*_* HB*: dead space fraction estimated using the unadjusted Harris-Benedict estimate of resting energy expenditure and the rearranged Weir equation for CO_2_ production, *CO*_*2*_* responders*: patients presenting a decrease of 1 mmHg or more in PaCO_2_ during proning, *PEEP*: positive end expiratory pressure, Pplat: plateau pressure, *∆P*: driving pressure, *C*_*RS*_: compliance of the respiratory system, p value refers to the comparison between the treatment success and treatment failure group

### Factors associated with treatment failure

Patient in the treatment failure group were older (73 [61–78] vs 60 [56–69] years, p = 0.009), had a higher SOFA score at admission (6 (3–8) vs 3 (2–4), p = 0.012), less frequently had a BMI > 30 kg/m^2^ (8 vs 47%, p = 0.03), and were less likely to be smokers (0% vs 33%, p = 0.04) (Table 2). In addition, patients in the treatment failure group were less frequently O_2_-responders to the first proning session (p = 0.002), but not to the second (p = 0.66) and third (p = 0.64). The patients in the treatment failure group showed a smaller variation in Aa-gradient during the first session (− 105 [− 169–− 13] mmHg vs − 170 [− 248–− 124] mmHg, p = 0.01), but no differences in the variations in VR, V_D_/V_T_ HB and C_RS_ during the first session (P = 0.21, p = 0.19, p = 0.13, respectively), compared to the patients in the treatment success group. Variations of the same parameters during the second and third sessions are presented in Table 3 for the patients with treatment success and failure. Response in terms of CO_2_ clearance was not associated to outcome for any of the first three sessions (p = 0.51, p = 0.45 and p = 0.77, respectively).

## Discussion

In this monocentric retrospective study performed in the Lausanne adult ICU during the first wave of the pandemic, we analyzed the physiological effects of prone positioning in a population of COVID-19 ARDS intubated patients treated with an early intubation, low to moderate PEEP and early proning strategy. At that time patients were managed in our ICU following a standardized protocol. They were systematically proned if they had a PaO_2_/FIO_2_ ratio < 150 mmHg despite curarization. Considering all proning sessions, we found a significative improvement in the PaO_2_/FiO_2_ ratio and reduction in Aa-gradient. We also found that 83% of the sessions lead to a more than 20% increase in the PaO_2_/FiO_2_ ratio. PaO_2_/FiO_2_ increment and Aa-gradient reduction was also found for each of the first five sessions individually. C_RS_ increased with proning when all the sessions were considered together and we noticed a slight increase in VR and V_D_/V_T_ HB. No serious adverse events were reported related to the proning sessions. Patients who experienced treatment failure were less frequently O_2_-responders to the first proning session, but not to the second and third sessions. They also had a smaller variation in the Aa-gradient following the first pronation. They were older, had a higher SOFA score at admission and were less frequently obese and smokers. Importantly, there were no differences in the time from intubation to the first proning, in the time from PaO_2_/FiO_2_ < 150 mmHg and the start of proning, in the number of proning sessions performed or in the sessions duration between the success and failure groups.

COVID 19 ARDS has some specificities compared to other forms of ARDS, such as severe hypoxemia being associated to slightly higher compliance in the early course of the disease [4, 34, 35]. In addition, according to Grieco et al. COVID-19 ARDS patients had higher VR, a dead space surrogate, than matched non COVID-19 patients [34]. This could be explained, even though still debated, to the presence of micro-thrombosis in the lung parenchyma [36], although technical issues such as dead space related to heat and moisture exchangers placed in the ventilator circuit might also be contributing factors [35]. In our cohort, we found a high VR at baseline shortly after intubation, in line with previous data in COVID patients [34, 37].

In the early phase of the pandemic, the value of the proning manoeuver in COVID-19 ARDS was questioned because of the above mentioned specificities [16], but the manoeuver has now been recognized as an adequate treatment option by experts [19].

Improvement in the PaO_2_/FiO_2_ ratio has been extensively reported during pronation in non-COVID-19 ARDS [9, 38] and the safety of this treatment strategy is well documented [38]. Increase in PaO_2_/FiO_2_ has been described in COVID-19 ARDS patients during proning [21–24, 28, 39] and our study confirms this finding. In addition, in our population treated with an early-intubation early-proning strategy, we found that a significant improvement in the PaO_2_/FiO_2_ ratio persists until the fifth proning session. This improvement is observed both with a reduction in FiO_2_ and a slight increase in PaO_2_. Several mechanisms may be implicated in this improvement in oxygenation. In ARDS from other causes, the main reason for the bettering of oxygenation is the improvement in ventilation-perfusion mismatch. This is a result of more homogeneous ventilation, with perfusion that is less altered by the prone position [13] and possibly less overdistension in the non-dependent lung regions. As a novel finding in our study, we observed the Aa-gradient to be practically halved at the end of the proning sessions. This observation was consistent throughout the five first sessions and definitely supports the major improvement of the ventilation to perfusion ratio during proning. This finding is of interest as this hypothesis is often mentioned as one of the main effects of prone positioning but very seldom reported in ARDS patients during proning.

Recruitment may in part account for the reduction in shunt fraction leading to better ventilation-perfusion matching. Indeed, in our cohort C_RS_ increased when considering all sessions collectively. Other papers reported variable results regarding changes in C_RS_ related to proning. Some showed improved compliance [27, 29], but others did not [21, 24]. The favorable effect of proning on C_RS_ was also shown in a small group of COVID-19 patients studied by electrical impedance tomography [40]. The change in C_RS_ is probably the result of both the change in thoracic compliance and in lung compliance due to the recruitment in the dorsal regions and compression in ventral regions [41] and this may differ in various patient cohorts. The favorable effects observed in our cohort might be due to proning in the early ARDS stage, in which lung may be more recruitable as suggested in the trial by Rossi et al. [42]. In this trial they found that during the first week of ventilation, patients with COVID-19 ARDS have more recruitable lung tissue than patients studied during the third week [42]. Interestingly, COVID-19 ARDS appears to be less recruitable than matched population with non-COVID-19 ARDS [43, 44]. It is worth noting that the change in C_RS_ was not significant if we consider each session independently. This could be explained by a heterogeneity of response to recruitment between patients and in the same patient at different timepoints as reported by Beloncle et al. [45].

C_RS_ might also fail to increase due to overdistention in non-dependent lung regions. This might be the case in our population as PEEP was not systematically adapted during proning and it was found to be only marginally decreased. In our cohort VR, a parameter which was correlated with measured V_D_/V_T_ in COVID-19 ARDS patients [46, 47] as well as in non-COVID ARDS, was only slightly increased with an amplitude that in our opinion is not clinically relevant and this may suggest that overdistention is not a major concern. The absence in a clinically significant change of dead space estimated by V_D_/V_T_ HB corroborates the absence of overdistention in our study.

Other studies investigated the changes in various physiological and respiratory parameters during proning in ARDS COVID-19 patients, but important differences between these studies and our must be underlined. Weiss and collaborators considered the first three proning sessions in 42 patients [24]. Their population could be compared to ours in term of C_RS_ and PaO_2_/FiO_2_, but their ventilation strategy differed with a higher set PEEP (median of 16 cmH_2_O compared to 12 in our study) and lower tidal volumes (median of 6 compared to 6.7 ml/kg of predicted body weight in our study). In contrast to our study, VR increased significantly and C_RS_ did not change during prone positioning in the Weiss’ study. The higher set PEEP strategy might have been responsible for increased overdistension and reduced perfusion in the overdistended area, as demonstrated in the physiological studies by Perier and Mauri [40, 48]. Our data reinforces the idea that a lower PEEP strategy might be beneficial in limiting overdistention, as suggested by some expert opinions [49] and recent data, at least in some COVID-19 ARDS patients [50]. Furthermore, differently from our population they observed a greater improvement in oxygenation for the first, second and third session in the treatment success group, whereas in our population, we found a major response (defined as an increase of at least 20% in the PaO_2_/FIO_2_ ratio) in terms of oxygenation only during the first proning session. Langer and collaborators studied 78 ARDS COVID-19 patients [21] before and after the first prone positioning. They found an improved PaO_2_/FiO_2_, but no difference in C_RS_ and VR. Ziehr et al. also studied the response to the first proning session in 122 patients with COVID-19. They found similar results compared to ours for the first session, with an improved PaO_2_/FiO_2_ ratio, but no change in C_RS_ or V_D_/V_T_ [39]. In addition to this, we can confirm with our data that the same findings apply for at least each of the first five sessions. As these studies only considered the first proning session, they do not answer the question whether the effects of proning in COVID-19 ARDS patients are temporary or persistent. In summary, our study is one of the few to address the effects of proning on as many physiological variables and on as many sessions in COVID-19 ARDS patients ventilated with a low to moderate PEEP strategy.

Regarding the outcome prediction in our study, we found a correlation between treatment failure defined as ICU death or need for VV-ECMO and older age, lower BMI and higher SOFA score at admission. Patients in the failure group were less frequently responders to the first proning session in terms of oxygenation. Naturally, our results regarding the factors associated with treatment success and failure must be confirmed in larger cohorts. Interestingly, a better response in terms of oxygenation to the first prone position was recently associated to lower mortality and weaning from mechanical ventilation at 28 days in a prospective study [51], which is congruent with our results.

The main limitation of this study is its retrospective and monocentric design. However, we assessed all the patients admitted during the study period and we examined a high number of prone position sessions. As important parameters were automatically recorded in the patients’ medical files, we had few missing data. Secondly, we studied patients who were treated according to an early-intubation, moderate PEEP and early-proning strategy. Our results cannot be generalized to other treatment strategies, for example for higher PEEP. It is worth noting that the median tidal volume in our patient cohort was 6.7 [6.0–7.0] ml/kg of predicted body weight (PBW), which is in line with values reported in the literature for patients treated during the first wave of COVID-19 [20] and with guidelines [11]. Whether our results on physiologic parameters would have been the same with lower tidal volumes is unknown. Thirdly, there was no control group. However, as proning is recommended by experts as being part of the management of COVID-19 ARDS with moderate to severe hypoxemia, having such a group is currently not possible. Fourthly, studying the effects of proning on advanced hemodynamic parameters, shunt fraction and right heart function would have been of interest. However, this was not feasible based on the available data as venous blood gases, central venous pressure measurements, invasive hemodynamic monitoring and echocardiography could not be systematically performed due to the workload during the pandemics. In practice, they were only performed in a minority of patients who were hemodynamically unstable. Fifthly, we must acknowledge that the threshold of 20% of improvement in PaO_2_/FIO_2_ ratio chosen to define significant improvement on oxygenation is arbitrary and that different results regarding the proportion of O_2_ responders could have been found with different thresholds. The threshold we used was the same as the threshold used in the Weiss et al. study [24] but differs from the threshold used in the Langer et al. [21] and in the Vollenberg et al. [26] studies who respectively used a fixed threshold of 20 mmHg and a 15% improvement in PaO_2_/FIO_2_ ratio. The threshold of decrease in 1 mmHg or more in PaCO_2_ used to assess CO_2_ response was also arbitrary, even if previously associated with improved outcome in non-COVID ARDS patients who were proned [33]. Finally, regarding the evaluation of the factors associated with treatment failure or success, it is important to underline that the composite outcome used to define treatment failure (ICU death or need of VV-ECMO) is arbitrary.

## Conclusion

In conclusion, our data showed the benefits of prone positioning in regards of improved oxygenation and reduction in alveolar-arterial gradient in COVID-19 ARDS patients with a PaO_2_/FiO_2_ ratio less than 150 mmHg treated with an early intubation, low to moderate PEEP and early pronation strategy. This was demonstrated for all proning sessions collectively as well as for the first and the subsequent sessions taken individually. When all the sessions were considered together, we also noticed an increase in C_RS_. No major complications related to proning were recorded. In addition, our data suggest that response to the first proning in terms of oxygenation and reduction of alveolar-arterial gradient might be linked to favorable outcome defined as survival without the need of VV-ECMO. Further studies are needed to confirm these interesting findings. From a clinical point of view, our study demonstrated similar physiological effects of proning in COVID-19 ARDS as compared to ARDS due to other causes and thus suggests that prone positioning in COVID-19 ARDS should be part of the standard of care.

## Supplementary Information

**Additional file 1: Table S1.** Physiological, ventilatory and hemodynamic data for all the sessions considered together (n=191) and for the first (n=42), the second (n=37), the third (n=31), the fourth (n=18) and the fifth (n=14) proning sessions). **Table S2.** Variations in physiological and ventilatory parameters during the fourth (n=18), the fifth (n=14) proning sessions. Comparisons between the success and failure group.

## Data Availability

The datasets used and/or analyzed during the current study are available from the corresponding author upon reasonable request.

## Abbreviations

Aa-gradient: Alveolo arterial gradient
ARDS: Acute respiratory distress syndrome
COVID19: Coronavirus disease 2019
C_rs_: Compliance of the respiratory system
EtCO_2_: End tidal partial pressure of carbon dioxide
FiO_2_: Fraction of inspired oxygen
PaCO_2_: Arterial partial pressure of carbon dioxide
PaO_2_: Arterial partial pressure of oxygen
PEEP: Positive end-expiratory pressure
PP: Prone position
Pplat: Plateau pressure
SAPS II: Simplified Acute Physiology Score II
SOFA: Sequential Organ Failure Assessment
VD/VT HB: Dead space fraction estimated using the unadjusted Harris-Benedict estimate of resting energy expenditure and the rearranged Weir equation for CO_2_ production
VILI: Ventilation induced lung injury
VR: Ventilatory ratio
