# Supplementary material for: Physiological response to prone positioning in intubated adults with COVID-19 acute respiratory distress syndrome: a retrospective study
Source: Respir Res. 2022 Nov 19;23:320. doi: 10.1186/s12931-022-02247-8 (PMC9675268; doi:10.1186/s12931-022-02247-8)
Supplement: Supplementary file 1 — Additional file 1: Table S1. Physiological, ventilatory and hemodynamic data for all the sessions considered together (n=191) and for the first (n=42), the second (n=37), the third (n=31), the fourth (n=18) and the fifth (n=14) proning sessions). Table S2. Variations in physiological and ventilatory parameters during the fourth (n=18), the fifth (n=14) proning sessions. Comparisons between the success and failure group. [file 12931_2022_2247_MOESM1_ESM.docx]

# Electronic supplementary material

# Additional file to the manuscript entitled “Physiological response to prone positioning in intubated adults with COVID-19 acute respiratory distress syndrome: a retrospective study”

Authors: Boffi Andrea ^1^, Ravenel Maximilien ^2^, Lupieri Ermes ^1^, Schneider Antoine ^1,2^, Liaudet Lucas ^1,2^, Chiche Jean-Daniel ^1,2^, Gonzalez Michel ^2,3^, Piquilloud Lise ^1,2^

Affiliations: ^1^ Department of Adult Intensive Care Unit, Lausanne University Hospital, Lausanne, Switzerland, ^2^ Faculty of Biology and Medicine, University of Lausanne, Lausanne, Switzerland, ^3^ Department of Thoracic Surgery, Lausanne University Hospital, Lausanne, Switzerland.

# Clinical protocol for the initial ventilator settings and PEEP setting

The clinical protocol to set the ventilator was the following.

- Patients were ventilated in volume assist control mode with an initial set tidal volume of 6 ml/kg of predicted body weight, an I:E ratio of 1:2, an inspiratory pause of 10% of the total inspiratory time and a respiratory rate titrated between 15 and 25/min in order to limit the hypercapnia without generating dynamic airtrapping.
- Initial PEEP was set according to the static respiratory system compliance (measured bedside by performing protocolized end-expiratory and end-inspiratory occlusions) according to the following rule. Initial PEEP was set between 10 to 12 cmH_2_O for patients with a respiratory system compliance of more than 35 ml/cmH_2_O and between 12 to 14 cmH_2_O in patients with a compliance lower than 35 ml/cmH_2_O. Plateau pressure monitoring was mandatory to keep it ≤ 28 cmH_2_O. It was also advised to target a maximal driving pressure of 14 cmH_2_O. If plateau pressure was higher than 28 cmH_2_O, a step by step decrease in PEEP was protocolized in order to find the highest possible PEEP while keeping plateau pressure ≤ 28 cmH_2_O.
- In case of pH <7.2 or PaCO_2_ >60 mmHg, respiratory rate was increased up to 35/min while checking for the absence of dynamic airtrapping. If this strategy did not allow to obtain pH > 7.2, then tidal volume was increased to 7-8 ml/kg and PEEP titrated again in order to maintain plateau pressure ≤ 28 cmH_2_O.
- During proning, PEEP was not systemically modified, but adaptations were made if requested using the same protocol as described above.
- At any time point, the senior ICU physician was allowed to adapt the ventilator settings based on his/her clinical evaluation. In practice, the usual strategy in our unit is to adapt PEEP according to the best compliance strategy and based on the hemodynamic effects.

- No recruitment maneuvers were performed except on very specific situations with clear derecruitment and only upon the senior ICU physician request.
- Weaning of PEEP by steps of 1 to 2 cmH_2_O was proposed in case of PaO_2_/FiO_2_ >200 mmHg after a minimum of 72h of protective ventilation.

# Table S1 Physiological, ventilatory and hemodynamic data for all the sessions considered together (n=191) and for the first (n=42), the second (n=37), the third (n=31), the fourth (n=18) and the fifth (n=14) proning sessions)

|  | Pre-PP | End-PP | P value |
| --- | --- | --- | --- |
| **Physiological variables** | | | |
| *PaO_2_, mmHg* | | | |
| All proning sessions | 64 (60-71) | 70 (65-77) | <0.001 |
| 1^st^ proning | 64 (59-72) | 73 (66-79) | <0.001 |
| 2^nd^ proning | 66 (61-71) | 73 (67-82) | 0.003 |
| 3^rd^ proning | 67 (63-71) | 73 (64-80) | 0.06 |
| 4^th^ proning | 64 (61-67) | 67 (64-76) | 0.06 |
| 5^th^ proning | 64 (59-70) | 71 (68-81) | 0.019 |
| *FiO_2_* | | | |
| All proning sessions | 0.60 (0.50-0.70) | 0.40 (0.35-0.50) | <0.001 |
| 1^st^ proning | 0.60 (0.55-0.70) | 0.40 (0.35-0.45) | <0.001 |
| 2^nd^ proning | 0.55 (0.45-0.64) | 0.40 (0.30-0.45) | <0.001 |
| 3^rd^ proning | 0.46 (0.4-0.65) | 0.40 (0.30-0.50) | <0.001 |
| 4^th^ proning | 0.70 (0.60-0.80) | 0.43 (0.35-0.46) | <0.001 |
| 5^th^ proning | 0.65 (0.60-0.71) | 0.45 (0.40-0.60) | <0.001 |
| *PaO_2_/FiO_2_, mmHg* | | | |
| All proning sessions | 107 (90-129) | 180 (148-210) | <0.001 |
| 1^st^ proning | 104 (94-126) | 186 (165-215) | <0.001 |
| 2^nd^ proning | 123 (103-151) | 197 (155-232) | <0.001 |
| 3^rd^ proning | 136 (97-161) | 200 (155-216) | <0.001 |
| 4^th^ proning | 93 (77-113) | 181 (148-215) | <0.001 |
| 5^th^ proning | 103 (85-112) | 168 (129-187) | <0.001 |
| *SaO2* | | | |
| All proning sessions | 92 (90-93) | 94 (92-95) | <0.001 |
| 1^st^ proning | 91 (90-93) | 94 (92-95) | <0.001 |
| 2^nd^ proning | 92 (91-94) | 94 (93-95) | 0.001 |
| 3^rd^ proning | 92 (91-94) | 94 (92-95) | 0.004 |
| 4^th^ proning | 91 (90-93) | 93 (92-95) | 0.012 |
| 5^th^ proning | 91 (90-94) | 94 (92-96) | 0.014 |
| *pH* | | | |
| All proning sessions | 7.38 (7.31-7.41) | 7.38 (7.33-7.42) | 0.032 |
| 1^st^ proning | 7.32 (7.26-7.39) | 7.36 (7.29-7.41) | 0.003 |
| 2^nd^ proning | 7.36 (7.30-7.40) | 7.36 (7.31-7.41) | 0.58 |
| 3^rd^ proning | 7.36 (7.31-7.41) | 7.37 (7.33-7.44) | 0.50 |
| 4^th^ proning | 7.33 (7.30-7.41) | 7.38 (7.30-7.42) | 0.22 |
| 5^th^ proning | 7.41 (7.30-7.43) | 7.38 (7.35-7.40) | 0.49 |
| *PaCO_2_, mmHg* | | | |
| All proning sessions | 56 (49-65) | 57 (50-64) | 0.86 |
| 1^st^ proning | 52 (48-62) | 53 (45-59) | 0.19 |
| 2^nd^ proning | 51 (48-58) | 52 (47-60) | 0.41 |
| 3^rd^ proning | 56 (48-64) | 58 (52-65) | 0.22 |
| 4^th^ proning | 64 (50-78) | 64 (57-68) | 0.67 |
| 5^th^ proning | 60 (56-68) | 57 (52-73) | 0.58 |
| *HCO_3_^-^, mmol/L* | | | |
| All proning sessions | 30.9 (26.8-36.7) | 32.5 (27.2-37.4) | 0.001 |
| 1^st^ proning | 27.2 (23.9-30.0) | 28.1 (24.5-31.3) | 0.026 |
| 2^nd^ proning | 29.1 (25.1-32.4) | 30.2 (25.8-34.1) | 0.11 |
| 3^rd^ proning | 30.3 (26.8-34.6) | 33.6 (28.4-36.8) | 0.002 |
| 4^th^ proning | 33.0 (27.4-39.1) | 34.2 (30.1-39.7) | 0.11 |
| 5^th^ proning | 34.3 (30.1-41.9) | 33.5 (30.2-42.0) | 0.92 |
| *Lactate, mmol/L* | | | |
| All proning sessions | 1.2 (0.9-1.4) | 1.2 (1.0-1.5) | 0.49 |
| 1^st^ proning | 1.0 (0.8-1.3) | 1.1 (1.0-1.4) | 0.029 |
| 2^nd^ proning | 1.1 (0.9-1.4) | 1.2 (1.0-1.5) | 0.10 |
| 3^rd^ proning | 1.3 (0.9-1.4) | 1.2 (1.1-1.4) | 0.47 |
| 4^th^ proning | 1.3 (1.0-1.5) | 1.3 (1.1-1.5) | 0.37 |
| 5^th^ proning | 1.4 (1.1-1.5) | 1.3 (0.9-1.5) | 0.24 |
| *EtCO2, mmHg* | | | |
| All proning sessions | 42 (38-47) | 43 (39 – 50) | 0.036 |
| 1^st^ proning | 42 (37-49) | 41 (33-50) | 0.15 |
| 2^nd^ proning | 43 (35-45) | 39 (34-48) | 0.95 |
| 3^rd^ proning | 43 (37-48) | 44 (40-51) | 0.07 |
| 4^th^ proning | 45 (37-54) | 46 (40-51) | 0.45 |
| 5^th^ proning | 42 (38-48) | 42 (39-48) | 0.21 |
| *PaCO_2_-EtCO_2_, mmHg* | | | |
| All proning sessions | 14 (9-23) | 13 (9-21) | 0.05 |
| 1^st^ proning | 12 (3-15) | 11 (6-15) | 0.85 |
| 2^nd^ proning | 13 (7-17) | 12 (7-19) | 0.63 |
| 3^rd^ proning | 17 (7-24) | 14 (9-22) | 0.55 |
| 4^th^ proning | 17 (10-29) | 13 (10-21) | 0.022 |
| 5^th^ proning | 42 (38-48) | 42 (39-48) | 0.21 |
| *Aa-gradient, mmHg* | | | |
| All proning sessions | 275 (211-334) | 127 (92-176) | <0.001 |
| 1^st^ proning | 276 (238-321) | 121 (89-160) | <0.001 |
| 2^nd^ proning | 240 (163-298) | 111 (79-164) | <0.001 |
| 3^rd^ proning | 196 (145-298) | 121 (72-165) | <0.001 |
| 4^th^ proning | 308 (278-401) | 135 (97-168) | <0.001 |
| 5^th^ proning | 295 (245-347) | 153 (125-227) | <0.001 |
| *VR* | | | |
| All proning sessions | 2.3 (1.9-2.8) | 2.4 (2.0-2.9) | 0.028 |
| 1^st^ proning | 2.1 (1.7-2.4) | 2.0 (1.7-2.5) | 0.55 |
| 2^nd^ proning | 2.0 (1.8-2.4) | 2.1 (1.7-2.5) | 0.18 |
| 3^rd^ proning | 2.2 (1.7-2.8) | 2.3 (1.9-2.7) | 0.14 |
| 4^th^ proning | 2.3 (2.0 -3.3) | 2.7 (2.1-3.2) | 0.21 |
| 5^th^ proning | 2.6 (2.1-3.7) | 2.5 (2.2-3.5) | 0.65 |
| *V_D_/V_T_ HB* | | | |
| All proning sessions | 0.71 (0.65-0.76) | 0.72 (0.67-0.76) | 0.022 |
| 1^st^ proning | 0.68 (0.62-0.73) | 0.68 (0.63-0.75) | 0.61 |
| 2^nd^ proning | 0.67 (0.61-0.71) | 0.69 (0.63-0.72) | 0.32 |
| 3^rd^ proning | 0.68 (0.61-0.77) | 0.71 (0.65-0.75) | 0.18 |
| 4^th^ proning | 0.74 (0.65-0.78) | 0.73 (0.70-0.78) | 0.15 |
| 5^th^ proning | 0.74 (0.70-0.83) | 0.74 (0.71-0.82) | 0.59 |
| **Ventilatory data** | | | |
| *Vt/PBW, ml/kg* | | | |
| All proning sessions | 6.9 (6.3-7.3) | 7.0 (6.3-7.4) | 0.002 |
| 1^st^ proning | 6.7 (6.3-7.3) | 6.9 (6.1-7.4) | 0.22 |
| 2^nd^ proning | 6.9 (6.1-7.2) | 6.9 (6.3-7.3) | 0.13 |
| 3^rd^ proning | 6.7 (6.4-7.3) | 6.9 (6.2-7.5) | 0.54 |
| 4^th^ proning | 7.0 (6.2-7.4) | 7.0 (6.1-7.6) | 0.33 |
| 5^th^ proning | 6.9 (6.1-7.6) | 7.1 (6.6-7.6) | 0.14 |
| *RR, min^-1^* | | | |
| All proning sessions | 23 (20-25) | 23 (21-26) | 0.08 |
| 1^st^ proning | 22 (20-25) | 22 (20-25) | 0.41 |
| 2^nd^ proning | 23 (20-25) | 22 (20-25) | 0.93 |
| 3^rd^ proning | 23 (20-25) | 23 (20-25) | 0.23 |
| 4^th^ proning | 24 (20-25) | 25 (20-26) | 0.049 |
| 5^th^ proning | 26 (21-27) | 25 (22-26) | 0.42 |
| *PEEP, cmH_2_O* | | | |
| All proning sessions | 12 (11-14) | 12 (11-13) | 0.027 |
| 1^st^ proning | 12 (11-14) | 12 (12-13) | 0.18 |
| 2^nd^ proning | 12 (10-13) | 12 (10-12) | 0.013 |
| 3^rd^ proning | 12 (10-14) | 12 (10-14) | 0.59 |
| 4^th^ proning | 12 (12-13) | 12 (12-14) | 0.047 |
| 5^th^ proning | 14 (12-15) | 13 (12-14) | 0.34 |
| *Pplat, cmH_2_O* | | | |
| All proning sessions | 27 (24-29) | 26 (24-28) | 0.002 |
| 1^st^ proning | 25 (23-27) | 24 (22-26) | 0.18 |
| 2^nd^ proning | 26 (23-28) | 26 (23-26) | 0.06 |
| 3^rd^ proning | 26 (22-27) | 25 (22-28) | 0.50 |
| 4^th^ proning | 28 (23-30) | 25 (24-31) | 0.50 |
| 5^th^ proning | 28 (26-30) | 27 (25-29) | 0.74 |
| *∆P, cmH_2_O* | | | |
| All proning sessions | 14 (12-16) | 14 (12-15) | 0.008 |
| 1^st^ proning | 13 (11-15) | 12 (10-14) | 0.24 |
| 2^nd^ proning | 14 (11-16) | 13 (11-14) | 0.26 |
| 3^rd^ proning | 12 (12-15) | 13 (11-14) | 0.31 |
| 4^th^ proning | 16 (12-18) | 13 (12-16) | 0.15 |
| 5^th^ proning | 14 (12-15) | 13 (12-14) | 0.34 |
| *C_RS_, ml/cmH_2_O* | | | |
| All proning sessions | 32.2 (27.5-40.9) | 36.2 (30.0-41.8) | 0.003 |
| 1^st^ proning | 34.3 (29.3-40.9) | 38.2 (32.9-44.2) | 0.23 |
| 2^nd^ proning | 32.7 (28.7-42.9) | 37.2 (32.1-42.2) | 0.12 |
| 3^rd^ proning | 36.8 (30.5-40.2) | 36.0 (30.8-47.1) | 0.43 |
| 4^th^ proning | 29.4 (27.3-41.3) | 35.6 (28.0-40.8) | 0.11 |
| 5^th^ proning | 32.7 (28.8-44.3) | 36.5 (31.3-41.3) | 0.92 |
| **Hemodynamics** | | | |
| *HR, min^-1^* | | | |
| All proning sessions | 83 (72-94) | 82 (73-97) | 0.42 |
| 1^st^ proning | 85 (75-96) | 80 (71-87) | 0.10 |
| 2^nd^ proning | 82 (72-89) | 78 (70-90) | 0.64 |
| 3^rd^ proning | 76 (65-86) | 84 (73-93) | 0.007 |
| 4^th^ proning | 84 (70-89) | 83 (74-97) | 0.71 |
| 5^th^ proning | 87 (76-104) | 83 (74-98) | 0.43 |
| *MAP, mmHg* | | | |
| All proning sessions | 77 (70-86) | 79 (71-88) | 0.23 |
| 1^st^ proning | 79 (71-85) | 75 (70-84) | 0.44 |
| 2^nd^ proning | 74 (68-85) | 79 (70-88) | 0.30 |
| 3^rd^ proning | 78 (68-87) | 81 (71-96) | 0.03 |
| 4^th^ proning | 75 (71-84) | 82 (74-86) | 0.18 |
| 5^th^ proning | 77 (72-100) | 81 (73-89) | 0.42 |
| *Norepinephrine, µg/kg/min* | | | |
| All proning sessions | 0 (0-0.04) | 0 (0-0.03) | 0.09 |
| 1^st^ proning | 0 (0-0.13) | 0 (0-0.09) | 0.57 |
| 2^nd^ proning | 0.03 (0-0.07) | 0 (0-0.04) | 0.08 |
| 3^rd^ proning | 0 (0-0.07) | 0 (0-0.09) | 0.15 |
| 4^th^ proning | 0 (0-0.02) | 0 (0-0.03) | 0.99 |
| 5^th^ proning | 0 (0-0.04) | 0 (0-0.01) | 0.13 |

**Table S1**

PaO_2_: arterial partial pressure of oxygen. FiO_2_: fraction of inspired oxygen. SaO_2_: arterial oxygen saturation. PaCO_2_: partial pressure of carbon dioxide. HCO_3_^-^: bicarbonate. EtCO_2_: end tidal CO_2._  Aa-gradient: alveolo-arterial gradient. VR: ventilatory ratio. V_D_/V_T_ HB: dead space fraction estimated using the unadjusted Harris-Benedict estimate of resting energy expenditure and the rearranged Weir equation for CO_2_ production. Vt: tidal volume. PBW: predicted body weight. RR: respiratory ratio. PEEP: positive end expiratory pressure. Pplat: plateau pressure. ∆P: driving pressure. C_RS_: compliance of the respiratory system. HR: heart rate. MAP: mean arterial pressure.

P value refers to the comparison between pre-PP and end-PP.

## Table S2 Variations in physiological and ventilatory parameters during the fourth (n=18), the fifth (n=14) proning sessions. Comparisons between the success and failure group.

|  | Overall | Success | Failure | P value |
| --- | --- | --- | --- | --- |
| *Variation in PaO_2_/FiO_2_, mmHg, from pre-PP to end-PP* | | | | |
| 4^th^ pronation | +81 (=37 - +115) | +89 (+34 - +89) | +58 (+32 - +116) | 0.39 |
| 5^th^ pronation | +65 (+36 - +90) | +77 (+53 - +97) | +47 (+23 - +81) | 0.22 |
| *Variation in FiO_2_ , from pre-PP to end-PP* | | | | |
| 4^th^ pronation | -0.30 (-0.35 - -0.24) | -0.28 (-0.35 - -0.21) | -0.30 (-0.35 - -0.22) | 0.89 |
| 5^th^ pronation | -0.20 (-0.27 - -0.10) | -0.23 (-0.29 - -0.20) | -0.15 (-0.23 - -0.05) | 0.13 |
| *Variation in Aa-gradient, mmHg, from pre-PP to end-PP* | | | | |
| 4^th^ pronation | -186 (-223 - -138) | -186 (-229 - -130) | -187 (-233 - -131) | 0.99 |
| 5^th^ pronation | -133 (-186 - -80) | -162 (-201 - -129) | -105 (-153 - -59) | 0.11 |
| *O_2_ responders* | | | | |
| 4^th^ pronation | 18/18 (100%) | 12/12 (100%) | 6/6 (100%) | 0.99 |
| 5^th^ pronation | 13/14 (93%) | 8/8 (100%) | 5/6 (83%) | 0.43 |
| *Variation in VR, from pre-PP to end-PP* | | | | |
| 4^th^ pronation | +0.08 (-0.20 - +0.43) | +0.07 (-0.18 - +0.58) | +0.08 (-0.31 - +0.40) | 0.57 |
| 5^th^ pronation | -0.03 (-0.23 - +0.28) | -0.06 (-0.22 - +0.24) | +0.02 (-0.26 - +0.34) | 0.85 |
| *Variation in V_D_/V_T_ HB, from pre-PP to end-PP* | | | | |
| 4^th^ pronation | +0.01 (-0.02 - +0.04) | +0.02 (-0.02 - +0.07) | +0.01 (-0.02 - +0.03) | 0.70 |
| 5^th^ pronation | 0 (-0.02 - +0.02) | -0.01 (-0.02 - +0.05) | +0.01 (-0.02 - +0.01) | 0.95 |
| *CO_2_ responders* | | | | |
| 4^th^ pronation | 11/18 (61%) | 6/12 (50%) | 5/6 (83%) | 0.17 |
| 5^th^ pronation | 9/14 (64%) | 6/8 (75%) | 3/6 (50%) | 0.33 |
| *Variation in PEEP, cmH_2_O, from pre-PP to end-PP* | | | | |
| 4^th^ pronation | 0 (0 - +2) | 0 (0 - +2) | 0 (0 - +2) | 0.99 |
| 5^th^ pronation | 0 (-1 – 0) | 0 (0 -0) | 0 (-1 – 0) | 0.41 |
| *Variation in Pplat, cmH_2_O, from pre-PP to end-PP* | | | | |
| 4^th^ pronation | 0 (-3 - +1) | -1 (-3 - +1) | +1 (-1 - +3) | 0.27 |
| 5^th^ pronation | 0 (-2 - +1) | -1 (-2 - +1) | 0 (-3 - +3) | 0.97 |
| *Variation in ∆P, cmH_2_O, from pre-PP to end-PP* | | | | |
| 4^th^ pronation | -1 (-3 - +4) | -1 (-4 - -1) | +1 (-1 - +1) | 0.09 |
| 5^th^ pronation | -1 (-1 - +1) | -1 (-2 - +1) | 0 (-3 - +4) | 0.76 |
| *Variation in C_RS_, ml/ cmH_2_O, from pre-PP to end-PP* | | | | |
| 4^th^ pronation | +3.1 (-0.9 - +6.7) | +4.1 (+1.6 - +9.6) | -0.9 (-2.1 - +2.4) | 0.08 |
| 5^th^ pronation | +1.1 (-2.9 - +4.8) | +1.8 (-5.3 - +6.1) | 0 (-13.0 - +8.0) | 0.79 |

**Table S2** PaO_2_: arterial partial pressure of oxygen. FiO_2_: fraction of inspired oxygen. Aa-gradient: alveolo-arterial gradient. O_2_ responders: patients presenting a 20% increase in PaO_2_/ FiO_2_ during proning. VR: ventilatory ratio. V_D_/V_T_ HB: dead space fraction estimated using the unadjusted Harris-Benedict estimate of resting energy expenditure and the rearranged Weir equation for CO_2_ production. CO_2_ responders: patients presenting a decrease of 1mmHg or more in PaCO_2_ during proning. PEEP: positive end expiratory pressure. Pplat: plateau pressure. ∆P: driving pressure. C_RS_: compliance of the respiratory system. P value refers to the comparison between the treatment success and treatment failure group.
